# Low Salicylic Acid Level Improves Pollen Development Under Long-Term Mild Heat Conditions in Tomato

**DOI:** 10.3389/fpls.2022.828743

**Published:** 2022-04-11

**Authors:** Stuart Y. Jansma, Lidiya I. Sergeeva, Yury M. Tikunov, Wouter Kohlen, Wilco Ligterink, Ivo Rieu

**Affiliations:** ^1^Plant Systems Physiology, Radboud Institute for Biological and Environmental Sciences, Radboud University, Nijmegen, Netherlands; ^2^Laboratory of Plant Physiology, Wageningen University and Research, Wageningen, Netherlands; ^3^Plant Breeding, Wageningen University and Research, Wageningen, Netherlands; ^4^Laboratory of Molecular Biology, Wageningen University and Research, Wageningen, Netherlands

**Keywords:** plant reproduction, *Solanum lycopersicum* (tomato), pollen development, salicylic acid, jasmonic acid, high temperature stress, transcriptome analysis (RNAseq), male fertility

## Abstract

Exposure to high temperatures leads to failure in pollen development, which may have significant implications for food security with ongoing climate change. We hypothesized that the stress response-associated hormone salicylic acid (SA) affects pollen tolerance to long-term mild heat (LTMH) (≥14 days exposure to day-/nighttime temperature of 30–34/24–28°C, depending on the genotype), either positively, by inducing acclimation, or negatively, by reducing investment in reproductive development. Here, we investigated these hypotheses assessing the pollen thermotolerance of a *35S:nahG* tomato line, which has low SA levels. We found that reducing the SA level resulted in increased pollen viability of plants grown in LTMH and further characterized this line by transcriptome, carbohydrate, and hormone analyses. Low expression of *JAZ* genes in *35S:nahG* and LTMH hypersensitivity of low-jasmonic acid (JA) genotypes together suggest that the increased pollen thermotolerance in the low-SA line involves enhanced JA signal in developing anthers in LTMH. These findings have potential application in the development of more thermotolerant crops.

## Introduction

Higher temperatures caused by climate change result in a significantly lower crop yield (Lobell and Field, 2007; Battisti and Naylor, 2009). In the second decade of the 21st century, the average global surface temperature has already increased by 1.09°C, relative to the estimated average global surface temperature during 1850–1900, and it is expected to increase further in the coming decades (Masson-Delmotte et al., 2021). This higher temperature results in an increase in the frequency, duration, and intensity of heatwaves (Masson-Delmotte et al., 2021).

Heat wave-type high-temperature stress [“long-term mild heat” (LTMH)] during flower development has been associated with a reduction in yield in many crop species (Charles and Harris, 1972; Munakata, 1976; Herrero and Johnson, 1980; Saini and Aspinall, 1982; Gibson and Paulsen, 1999; Angadi et al., 2000; Sato et al., 2002; Wang et al., 2006). In particular, a strong correlation has been found between fruit-set or crop yield and pollen viability (PV) (Sakata et al., 2000; Peng et al., 2004; Prasad et al., 2006; Farooq et al., 2011; Song et al., 2015; Xu et al., 2017; Driedonks et al., 2018; Wu et al., 2021).

Previous studies have identified thermosensitive stages of pollen development and provided some hypotheses for the mechanisms behind LTMH-induced pollen abortion, including altered phytohormone signaling (De Storme and Geelen, 2014), loss of tapetum identity in the anthers (Parish and Li, 2010; Müller et al., 2016), desynchronization of tapetum and pollen development (Suzuki et al., 2001; Ku et al., 2003; Endo et al., 2009; Parish et al., 2012; Meng et al., 2016; Yu et al., 2016), and disturbance of carbohydrate metabolism (Pressman et al., 2002). It remains to be elucidated whether acclimation responses take place in the anther to protect pollen development against LTMH and whether LTMH-induced pollen failure is the passive result of heat injury, or the result of an active cell physiological response to heat stress (Müller and Rieu, 2016).

Salicylic acid (SA) is involved in a range of acclimation responses, also in those to high temperatures, especially in case of heat shock. This type of heat stress has been shown to lead to the accumulation of SA in *Arabidopsis* seedlings (Clarke et al., 2009) and seedlings with reduced SA levels or signaling are more sensitive to heat shock (Larkindale and Knight, 2002; Clarke et al., 2004). Conversely, treatment with SA preceding heat exposure improved basal thermotolerance of Arabidopsis seedlings (Clarke et al., 2009). SA treatment has also been shown to have a positive effect on the thermotolerance of vegetative tissues in wheat (Khan et al., 2013). Furthermore, a single treatment with SA, prior to the onset of long-term exposure to 40°C, improved pollen thermotolerance in rice and this was linked to an increase in reactive oxygen species (ROS) scavenging, attenuation of Caspase 3 activity, and timely tapetum degradation (Zhang et al., 2017; Feng et al., 2018).

Acclimation responses are costly, meaning that trade-offs with growth and developmental processes are to be expected. A well-studied example is a trade-off between growth/development and defense (Herms and Mattson, 1992; Huot et al., 2014). Reducing the biotic stress response allows for more allocation of resources to growth and development (Simms and Rausher, 1987; Campos et al., 2016) and hormone signals that regulate growth and development, such as auxin (IAA), brassinosteroids, and gibberellin, may be inhibited by stress hormone signaling, such as from SA (Huot et al., 2014; Verma et al., 2016). We hypothesized that SA plays a role in tomato pollen LTMH tolerance, either in a positive sense by inducing a protective acclimation response, or in a negative sense, by reducing investment in pollen development under heat stress. To this end, we examined the effects of reduced SA level on tomato pollen thermotolerance and physiology and further characterized the pollen LTMH tolerance of the *35S:nahG* low-SA genotype.

## Materials and Methods

### Plant Material and Cultivation

*Solanum lycopersicum 35S:nahG* has been described before (Brading et al., 2000) and was introgressed into cv. Micro-Tom (Carvalho et al., 2011). *def1* and *acx1* were in the Castlemart background (Howe et al., 1996; Li et al., 2005). Plants were grown on potting soil (No. 4, Horticoop, Lentse Potgrond, Slingerland Potgrond) with 4 g/L Osmocote Exact Standard 3–4 M (Everris). Plants were grown in climate-controlled cabinets with a 12/12 h photoperiod (Philips Green Power LED DR/B/FR 120, ∼250 μmol m^–2^s^–1^ at plant height) at 25°C during the daytime and 19°C during the nighttime and 50% relative humidity (RH). For LTMH treatment, plants were moved into LTMH conditions upon flowering. LTMH consisted of daytime temperature of 30–34°C and a corresponding 6°C lower night temperature, dependent on the experiment. RH in LTMH was adjusted to maintain equal vapor pressure deficit (VPD) as compared to control temperature (CT) conditions.

### Pollen Viability Measurements

Flowers at the anthesis stage were sampled in CT conditions or after 10–21 days in LTMH conditions, on at least 3 different days. PV was determined through impedance flow cytometry, using Ampha™ Z30 with D-chip, AF6 buffer, and other settings as recommended for tomato by the manufacturer (Amphasys AG, Root, Switzerland) (Heidmann et al., 2016). On each sampling day, 1–4 flowers at the stage of anthesis of each plant were pooled into a sample, and viability was determined for ∼20.000 pollen per sample.

### Sampling and RNA Extraction for RNA-Seq

Anthers with polarized stage microspores (4.6–4.8 mm), as determined by DAPI staining of the pollen, were collected from CT and after 8 days of exposure to LTMH conditions. Samples were taken approximately 5 h into the light period. Six anthers were pooled per replicate and 4 biological replicates were generated for both the conditions and genotypes. RNA was isolated *via* TRI Reagent (Sigma-Aldrich, MA, United States) extraction with cleanup on the Qiagen Plant RNeasy (catalog no. 74104) columns. All the RNAs were treated with DNaseI using the Plant RNeasy Kit. RNA degradation and contamination were monitored on 1.5% agarose gels. RNA purity was checked using the NanoPhotometer^®^ spectrophotometer (IMPLEN, CA, United States). RNA integrity and quantitation were assessed using the RNA Nano 6000 Assay Kit of the Bioanalyzer 2100 System (Agilent Technologies, CA, United States).

### Ribonucleic Acid Sequencing

A total amount of 1 μg RNA per sample was used as input material for the RNA sample preparations. Sequencing libraries were generated using the NEBNext^®^ Ultra™ RNA Library Prep Kit for Illumina^®^ (NEB, United States) following the manufacturer’s recommendations and index codes were added to attribute sequences to each sample. Briefly, messenger RNA (mRNA) was purified from total RNA using poly-T oligo-attached magnetic beads. Fragmentation was carried out using divalent cations under elevated temperature in NEBNext First Strand Synthesis Reaction Buffer (5X). First-strand cDNA was synthesized using random hexamer primer and MMuLV Reverse Transcriptase (RNase H-). Second strand cDNA synthesis was subsequently performed using DNA polymerase I and RNase H. Remaining overhangs were converted into blunt ends *via* exonuclease/polymerase activities. After adenylation of 3′ ends of DNA fragments, NEBNext Adaptor with hairpin loop structure was ligated to prepare for hybridization. To select cDNA fragments of preferentially 150–200 bp in length, the library fragments were purified with the AMPure XP system (Beckman Coulter, Beverly, MA, United States). Then, 3 μl USER Enzyme (NEB, United States) was used with size-selected, adaptor-ligated cDNA at 37°C for 15 min followed by 5 min at 95°C before PCR. Then PCR was performed with Phusion High-Fidelity DNA polymerase, Universal PCR primers, and Index (X) Primer. At last, PCR products were purified (AMPure XP system) and library quality was assessed on the Agilent Bioanalyzer 2100 System.

The clustering of the index-coded samples was performed on a cBot Cluster Generation System using PE Cluster Kit cBot-HS (Illumina) according to the manufacturer’s instructions. After cluster generation, the library preparations were sequenced on an Illumina platform and 125/150 bp paired-end reads were generated. Raw reads of fastq format were filtered to remove reads containing adapters or reads of low quality, i.e., when uncertain nucleotides constitute more than 10% of either read and when low-quality nucleotides constitute more than 50% of the read. All the downstream analyses were based on clean data. Index of the reference genome (ITAG3.2) was built using Bowtie version 2.2.3 and paired-end clean reads were aligned to the reference genome using hisat2 version 2.0.5, using the following parameters: –dta –phred33. In total, 94.7% of the reads were uniquely mapped, of which for each sample at least 90% were exon-mapped reads. FeatureCounts v1.5.0-p3 software was used to count the reads numbers mapped to each gene, using default parameters. Fragments per kilobase of exon per million mapped fragments (FPKM) of each gene was calculated based on the length of the gene and reads count mapped to this gene, using a threshold FPKM of 0.1. Differential expression analysis of two conditions/groups was performed using the DESeq R package (1.18.0). The resulting *P*-values were adjusted using Benjamini and Hochberg’s approach for controlling the false discovery rate (FDR). Genes with an FDR adjusted *P*-value < 0.05 found by DESeq and —foldchange— ≥ 1.5 were assigned as differentially expressed.

### Transcriptome Analyses

Principal component analysis (PCA) was performed using the online tool ClustVis^1^ with all the standard settings (i.e., row centering on, row scaling with unit variance, and singular value decomposition with imputation). Genes were included if at least one sample had an FPKM value of at least 1. Unit variance scaling was applied. Prediction ellipses are such that with a probability of 0.95, a new observation from the same group will fall inside the ellipse. *n* = 3 or 4 samples (PC1, principal component 1; PC2, principal component 2).

A transcriptome-wide gene set enrichment analysis (FDR *q* < 0.05) for gene ontology (GO)-slim biological process annotations was performed on ranked fold-changes using the online software tool Panther (version 16.0^2^) with FDR correction (Mi et al., 2021). Over-representation of GO-slim biological process annotations among up- or downregulated genes was also determined using Panther, with Fisher’s exact test and FDR correction. Targeted overrepresentation analyses were performed by calculating the statistical significance of the overlap between differentially expressed genes (DEGs) within a gene set and all DEGs among the whole genome using a Chi-square test with Yates’ correction. The gene sets for pollen development and carbohydrate metabolism and transport were generated based on homology to annotated Arabidopsis genes, the gene set for heat shock response (HSR) was based on Fragkostefanakis et al. (2015) and Keller et al. (2018), and the gene sets for hormone metabolism, signaling, and response were derived from the annotation of the tomato genome version SL3.0 ITAG3.2 by Biobam using Omicsbox (Biobam, Valencia, Spain) (Supplementary Table 1).

### Sampling for Carbohydrate and Hormone Measurements

Pollen stage was linked to flower length by categorizing 4′,6-diamidino-2-phenylindole (DAPI)-stained pollen and stages were selected based on their synchronized states when grown at different temperature regimes (Supplementary Table 2). Anthers were immediately frozen in liquid nitrogen upon isolation, and stored at –80°C.

### Extraction and Quantification of Carbohydrates

Soluble carbohydrates were extracted by homogenizing 100 mg frozen anther material in 300 μl MeOH. The sample was diluted with 330 μl methanol and 300 μl water to 1 ml 70% methanol. After shaking for 30′ at RT, the sample was centrifuged at max speed. In total, 500 μl supernatants was transferred to a new Eppendorf tube, and 450 μl water and 250 μl chloroform were added. After vortexing for 5′, the sample was centrifuged at max speed. In total, 200 μL supernatant was transferred to a new Eppendorf tube and dried in a speed-vac. Samples were then analyzed using a Dionex HPLC system (Dionex, Sunnyvale, CA, United States), and analyzed using a CarboPac PA100 4 mm × 250 mm column followed by a guard column (CarboPac PA100, 4 mm × 50 mm), a gradient pump module (model GP40) and an ED40-pulsed electrochemical detector. Mono-, di-, and trisaccharides were separated by elution in an increasing concentration of NaOH (50–200 mM) with a flow rate of 1 ml min^–1^. Peaks were identified by the co-elution of standards. The final sugar quantity was corrected *via* the internal standard (IS) and transformed to micrograms of sugar per milligram of fresh weight.

### Extraction of ABA, SA, IAA, JA-ile, and CKs (iP, tZ, and cZ)

For extraction of salicylic acid (SA), abscisic acid (ABA), auxin (IAA), jasmonic acid-isoleucine (JA-ile), and cytokinins (CKs: iP, isopentyladenine; tZ, *trans*-zeatin; cZ, *cis*-zeatin), 70 mg of snap-frozen anther tissue was used per sample. The tissue was ground to a fine powder at –80°C using 3-mm stainless steel beads at 50 Hz for 1 min in a TissueLyser LT (Qiagen, Germantown, MD, United States). Ground samples were extracted with 1 ml of 100% methanol (MeOH) containing stable isotope-labeled IS (Supplementary Table 3). IS were used at an end concentration of 100 nM per compound per sample. Samples were extracted as previously described (Schiessl et al., 2019; Gühl et al., 2021) with the addition that, prior to the elution of CKs, acid phytohormones were eluted with 1 ml of 100% MeOH. All solvents were evaporated in a speed vacuum system (SPD121P, ThermoSavant, Hastings, United Kingdom) at room temperature and the residue stored at –20°C until further analysis.

### Detection and Quantification of IAA, JA-ile, iP, tZ, and cZ by LC-MS/MS

Sample residues were dissolved in 100 μL of acetonitrile/water (acid hormones) or MeOH/water (CKs) (0.1% formic acid) (20:80, v/v), and filtered through a 0.45 mm Minisart SRP4 filter (Sartorius, Goettingen, Germany). Analyses of plant growth regulators were performed by comparing retention times and mass transitions with those of unlabeled standards (Supplementary Table 3) using a Waters XevoTQs mass spectrometer equipped with an electrospray ionization source coupled to an Acquity UPLC system (Waters, Milford, CT, United States). Chromatographic separations were conducted on an Acquity UPLC BEH C18 column (100 mm, 2.1 mm, 1.7 mm; Waters, United States) by applying an acetonitrile/water (0.1% formic acid) or methanol/water (0.1% formic acid) gradient. The column was operated at 50°C with a flow rate of 0.5 ml min^–1^. The column was equilibrated for 30 min using either solvent composition at the start of a run. The acetonitrile/water (0.1% formic acid) gradient started from 20% (v/v) acetonitrile, increasing to 70% (v/v) acetonitrile in 17 min. To wash the column, the water/acetonitrile gradient was increased to 100% (v/v) acetonitrile in a 1.0 min gradient, which was maintained for 1.0 min before going back to 20% acetonitrile using a 1.0 min gradient, prior to the next run. The methanol/water (0.1% formic acid) gradient started from 5% (v/v) methanol, increasing to 70% (v/v) methanol in 17 min. To wash the column, the water/methanol gradient was increased to 100% (v/v) methanol in a 1.0 min gradient, which was maintained for 1.0 min before going back to 5% methanol using a 1.0 min gradient, prior to the next run. The sample injection volume was 3/5 μl (acids/CK, respectively). The mass spectrometer was operated in positive and negative electrospray ionization mode when required. Cone and desolvation gas flows were set to 150 and 1,000/800 l h^–1^, respectively. The capillary voltage was set at 3.5/3.0 kV, the source temperature at 150°C, and the desolvation temperature at 550°C. The cone voltage was optimized for each standard compound using the IntelliStart MS Console (Waters, Milford, CT, United States). Argon was used for fragmentation by collision-induced dissociation. Multiple reaction monitoring (MRM) was used for quantification (Gühl et al., 2021). Parent–daughter transitions for the different (stable isotope-labeled) compounds were set using the IntelliStart MS Console. MRM transitions, cone voltage, and collision energy selected for compound identification and quantification are shown in Supplementary Table 3. To determine sample concentrations, a 10-point calibration curve was constructed for each compound ranging from 1 μM to 190 pM and each dilution also contained a known amount of an appropriate deuterium-labeled IS.

### Statistics

Pollen viability was averaged for each plant and logit transformed using the formula PV* = LN [(PV + 1)/(101 − PV)] for statistical analysis. Carbohydrate and phytohormone concentrations were log10 transformed for statistical analysis. Statistical significance was tested by means of a one-way ANOVA with LSD or Games–Howell *post hoc* analysis, as appropriate, using IBM SPSS Statistics version 25.

## Results

### Endogenous Salicylic Acid Reduces Pollen Long-Term Mild Heat Tolerance

**FIGURE 1:**
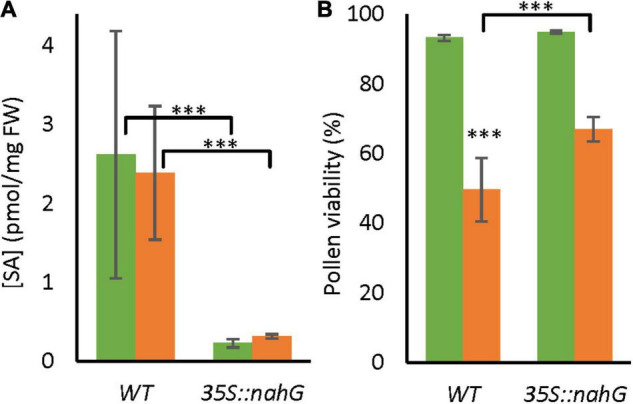
Endogenous salicylic acid (SA) levels and pollen long-term mild heat (LTMH) tolerance of WT and *35S:nahG*. Green bars represent control temperature (CT), orange bars LTMH. **(A)** SA levels in bicellular pollen stage anthers of wild type (WT) and *35S:nahG* after exposure to LTMH. LTMH consisted of repeated exposure to 34°C (day) and 28°C (night). Hormone concentrations were measured after 7 days of exposure to the treatment. Values indicate the mean ± SD (*n* = 6 samples, with pools of 20 anther cones per sample). Two-way ANOVA main effects: temperature ns; genotype *P* < 0.001; interaction ns. **(B)** Pollen viability of WT and *35S:nahG* after exposure to LTMH. LTMH consisted of repeated exposure to (33.5°C day and 27.5°C night). Pollen viability was measured 14–20 days after the onset of the treatment. Values indicate the mean ± SD (*n* ≥ 4 plants, with multiple pools of 3 anther cones per plant). Two-way ANOVA main effects: temperature *P* < 0.001; genotype *P* < 0.001; interaction *P* < 0.05. *Significantly different between LTMH and CT in WT or between mutant and WT within temperature treatment (brackets), one-way ANOVA with LSD *post hoc*, *P* < 0.05; ^**^P < 0.01; ^***^P < 0.001.

To test whether the endogenous SA signal affects the tolerance of pollen development to LTMH, we analyzed a transgenic line with constitutive expression of a bacterial gene that leads to SA degradation, *35S:nahG*, grown in LTMH conditions from the start of flowering. SA level of bicellular-stage anthers did not significantly change after exposure to LTMH in wild type (WT) or *35S:nahG*, but, as expected, the *35S:nahG* line contained significantly less SA than the WT (Figure 1A). Pollen from wild-type flowers that had fully developed in LTMH had significantly lower viability than those from the CT treatment (Figure 1B and Supplementary Figure 1). In the low-SA line, PV was not different from WT in CT, but significantly higher than WT in LTMH (Figure 1B).

### Transcriptome Changes in the Long-Term Mild Heat Tolerant Low-Salicylic Acid Line

**FIGURE 2:**
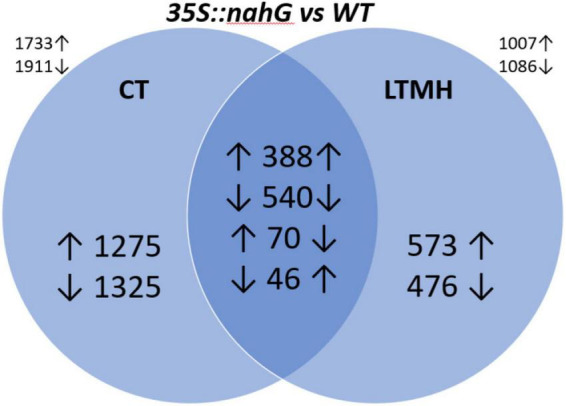
Differentially expressed genes (DEGs) between polarized stage anthers of WT and *35S:nahG* in CT and LTMH. LTMH consisted of repeated exposure to 34°C (day) and 28°C (night). Values indicate the number of DEGs, and up and down arrows indicate higher or lower expression, respectively, in *35S:nahG*. An arrow left to the value indicates the direction of response in CT; on the right, in LTMH. The values outside the diagram indicate the total number of upregulated and downregulated DEGs for the concerning temperature condition.

To get insight into the physiology behind pollen LTMH tolerance, we compared gene expression levels of developing anthers of *35S:nahG* and the wild type (Supplementary Table 4). Principle component analysis of the gene expression levels showed that the LTMH treatment had the biggest influence on gene expression, followed by genotype (Supplementary Figure 2). Two-thirds of the genes that responded to LTMH in WT (|FC| > 1.5; FDR *q* < 0.05), responded similarly in *35S:nahG*, but in the latter, the response was more extensive with about 50% more genes responding in total (Supplementary Figure 3). In total, 1,007 genes were found to be higher and 1,086 genes were found to be lower expressed in the low-SA line than in WT in LTMH (Figure 2).

**FIGURE 3:**
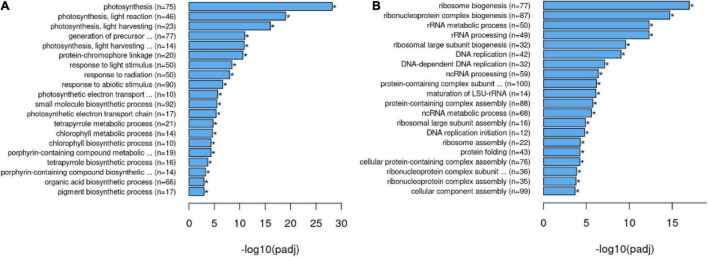
Overrepresentation analysis of GO biological processes among genes differentially expressed between *35S:nahG* and WT in LTMH. Top categories are indicated among **(A)** DEGs that are higher expressed in *35S:nahG* than in WT (Supplementary Table 6) and **(B)** DEGs that are lower expressed in *35S:nahG* than in WT (Supplementary Table 7).

To obtain a broad overview of processes modified in the *35S:nahG* line in LTMH, a transcriptome-wide gene set enrichment analysis (FDR *q* < 0.05) for GO-slim biological process annotations was performed. This showed a bias toward a higher expression of genes related to photosynthesis, response to ABA, generation of precursor metabolites, and energy and glucose metabolism (Supplementary Table 5). This was largely mirrored in the overrepresentation analysis among upregulated genes, with photosynthesis-related GO terms as the most upregulated sets, in addition to the response to abiotic stress and glucose and fructose metabolism (Figure 3A and Supplementary Table 6). Enrichment and overrepresentation analyses revealed lower expression of genes related to mitosis, ribosomal RNA/translation, response to endoplasmic reticulum (ER) stress, response to unfolded protein, as well as response to wounding/regulation of defense response (Figure 3B and Supplementary Tables 5, 7). Notably, the downward trend of response to wounding and regulation of defense response was mostly caused by genes encoding JAZ proteins, repressors in the JA signal transduction pathway, being less upregulated by LTMH, while no change was observed in the expression of the COI1 JA receptor gene (Supplementary Figure 4).

To better understand the differences between *35S:nahG* and WT in the expression of genes related to pollen development, the heat shock response, carbohydrate metabolism and transport, and hormone physiology, we performed targeted overrepresentation analyses with corresponding gene sets (Supplementary Table 8). A number of pollen development-related genes was expressed either higher or lower in the low-SA line in LTMH, although not more than expected. In particular, *MPK3* and *CALS5* homologs were expressed much higher in the low-SA line in LTMH (i.e., around 4–5-fold), whereas *WBC27*, *TDF1*, *CYP704B1*, and *CYP703A2* were expressed much lower (10–37-fold; Supplementary Table 9). The lower expression of *TDF1* was already seen in CT. Similarly, a number of HSR genes was expressed differentially between *35S:nahG* and WT in LTMH, but the total did not deviate significantly from what was expected. HSR-related differences included 2.5–3.5-fold higher expression of three HSF genes (two *HSFA6b* and *HSF9*) and a mitochondrial HSP40 gene, and strongly lower expression (40- to 225-fold) of genes for two small-HSPs and an HSP40. Most of these differences were already observed in CT conditions (Supplementary Table 10). Concerning carbohydrate-related genes, the amplitude of the higher expressed metabolic genes was limited (around twofold) and included a variety of enzymes. Three genes were more clearly lower expressed (3- to 9-fold) and included two types of invertases (vacuolar and cell wall) as well as an invertase inhibitor (Supplementary Table 11). Among the carbohydrate transport genes, the hexose transporters *SlSTP6* and *SlSWEET5a* were expressed much higher in the low-SA line in LTMH (around 15-fold). Several other SWEET transporter genes were lower expressed (2- to 4-fold; Supplementary Table 12). Finally, we analyzed genes related to metabolism, signaling, and response of several hormones (Supplementary Table 13). A significant number of ABA response, auxin signaling, and ethylene metabolism genes were overrepresented among genes that were expressed higher in *35S:nahG*. This included the orthologs of ABA-responsive *AtLTI65* and an LEA protein (6- to 37-fold), both involved in the regulation of water balance, an ACC-synthase, and two ACC-oxidases (2- to 3-fold) and several AUX/IAA-like repressors of auxin signaling (1.5 to 3-fold). There was, however, no clear overall pattern in expression changes of the TIR and AUX/IAA signaling component genes between *35S:nahG* and WT in LTMH, with both upregulated and downregulated members (Supplementary Figure 5).

### Carbohydrate Content in the Long-Term Mild Heat-Tolerant Low-Salicylic Acid Line

**FIGURE 4:**
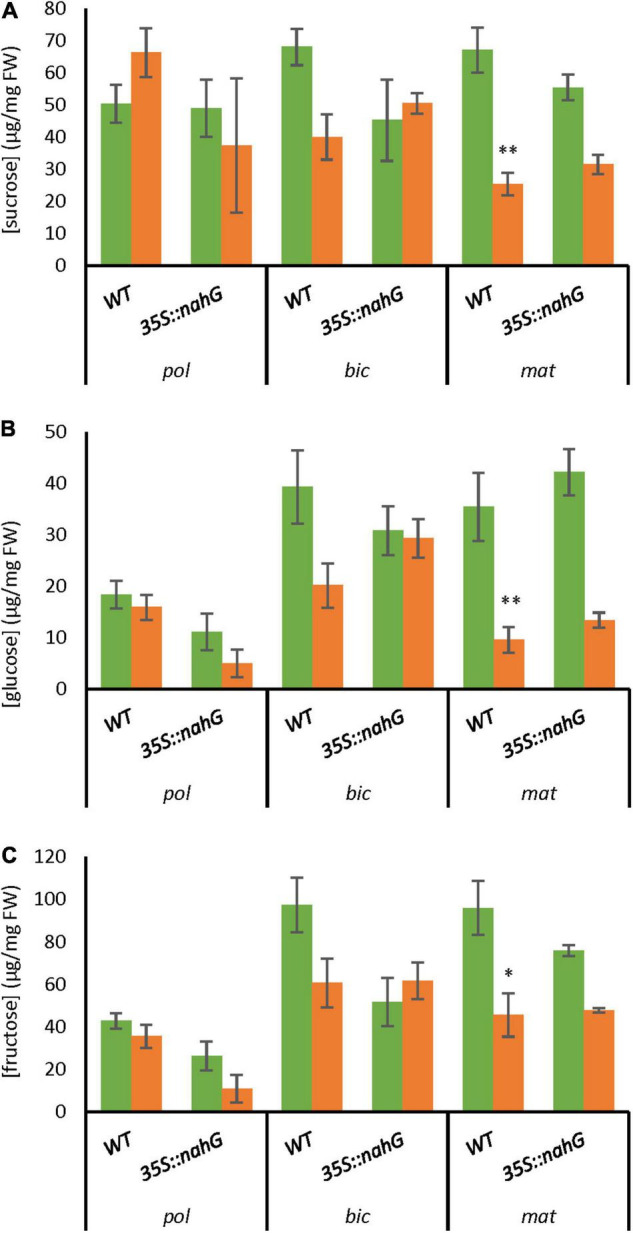
Simple carbohydrate levels in developing anthers of WT and *35S:nahG* in CT and LTMH. LTMH consisted of repeated exposure to 34°C (day) and 28°C (night). Carbohydrate concentrations were measured after 4, 7, and 10 days of exposure to the treatment, respectively, for polarized microspore (pol), bicellular pollen (bic), and mature pollen (mat) stage anthers. Green bars represent CT, orange bars LTMH. Values indicate the mean ± SD (*n* = 6 samples, with pools of 20 (pol), 15 (bic), or 10 (mat) anther cones per sample). **(A)** Sucrose levels. Two-way ANOVA main effects in pol/bic/mat: temperature *P* < 0.05/ < 0.01/ < 0.001; genotype ns/*P* < 0.05/ns; interaction ns/*P* < 0.05/ < 0.01. **(B)** Glucose levels. Two-way ANOVA main effects in pol/bic/mat: temperature ns/*P* < 0.01/ < 0.001; genotype *P* < 0.01/ns/*P* < 0.05; interaction ns/*P* < 0.01/ns. **(C)** Fructose levels. Two-way ANOVA main effects in pol/bic/mat: temperature ns/ns/*P* < 0.01; genotype *P* < 0.01/*P* < 0.01/ns; interaction ns/*P* < 0.01/ns. *Significantly different within stage between LTMH and CT in WT or between mutant and WT within temperature treatment (brackets), one-way ANOVA with Games–Howell *post hoc*, *P* < 0.05; ^**^P < 0.01; ^***^P < 0.001.

Given the observed differences in carbohydrate-associated gene expression between the low-SA line and the wild type in LTMH, we investigated potential changes in carbohydrate levels in developing anthers when exposed to LTMH. In the WT at the polarized micropore stage, an opposite LTMH trend was seen for sucrose (up) and the hexoses (down). At the bicellular pollen stage, there was a downward trend for all sugars, and this LTMH response became statistically significant at the mature pollen stage. Differences between *35S:nahG* and WT were rather limited in either condition. In LTMH, there was a trend toward lower hexose content in polarized microspore stage anthers, slightly higher glucose levels at bicellular stage anthers, as well as slightly higher sucrose and glucose levels at mature pollen stage anthers (Figure 4).

### Hormone Levels in the Long-Term Mild Heat-Tolerant Low-Salicylic Acid Line

**FIGURE 5:**
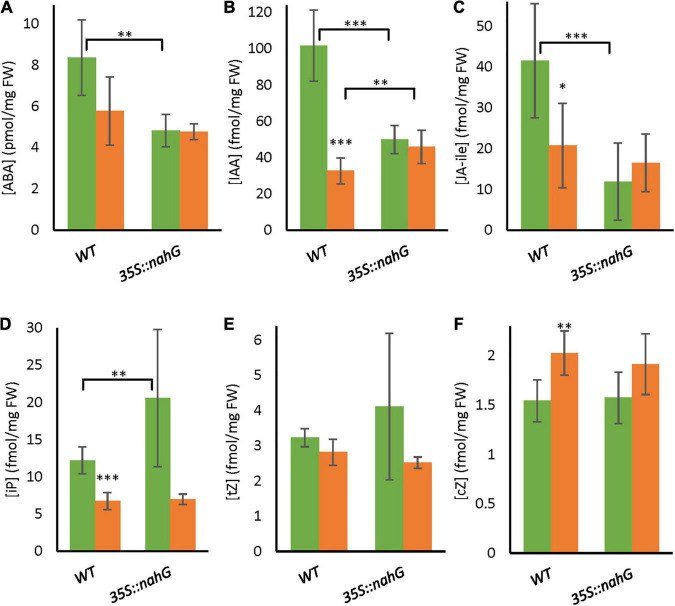
Hormone levels in bicellular pollen stage anthers of WT and *35S:nahG* after exposure to LTMH. LTMH consisted of repeated exposure to 34°C (day) and 28°C (night). Hormone concentrations were measured after 7 days of exposure to the treatment. Green bars represent CT, orange bars LTMH. Values indicate the mean ± SD (*n* = 6 samples, with pools of 20 anther cones per sample). **(A)** Abscisic acid (ABA) levels. Two-way ANOVA main effects: temperature *P* < 0.05; genotype ns; interaction *P* < 0.05. **(B)** IAA levels. Two-way ANOVA main effects: temperature *P* < 0.001; genotype *P* < 0.05; interaction *P* < 0.001. **(C)** Jasmonic acid-isoleucine (JA-ile) levels. Two-way ANOVA main effects: temperature ns; genotype *P* < 0.01; interaction *P* < 0.01. **(D)** iP levels. Two-way ANOVA main effects: temperature *P* < 0.001; genotype *P* < 0.05; interaction *P* < 0.05. **(E)** tZ levels. Two-way ANOVA main effects: temperature *P* < 0.01; genotype ns; interaction ns. **(F)** cZ levels. Two-way ANOVA main effects: temperature *P* < 0.01; genotype ns; interaction ns. *Significantly different between LTMH and CT in WT or between mutant and WT within temperature treatment (brackets), one-way ANOVA with Games–Howell or LSD *post hoc*, *P* < 0.05; ^**^P < 0.01; ^***^P < 0.001.

To further investigate potential interactions between SA and other plant hormones, we measured the level of ABA, JA-ile, IAA, and various CKs in bicellular-stage anthers of *35S:nahG* and the wild type (Figure 5). There was a trend toward lower ABA content in anthers of the wild type when exposed to LTMH, and this reduction was significant for IAA, JA-ile, and iP, while the concentration of cZ was increased. In CT, *35S:nahG* had lower levels of ABA, IAA, and JA-Ile than WT, and a higher level of iP. The only difference between *35S:nahG* and WT in LTMH was a slight increase in IAA level (Figure 5B).

### Jasmonic Acid Is Required for Pollen Long-Term Mild Heat Tolerance

**FIGURE 6:**
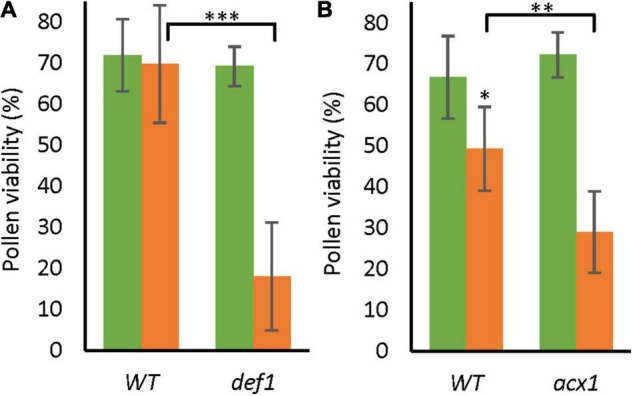
Pollen viability of low-JA genotypes in CT and LTMH conditions. Pollen viability was measured 14–20 days after the onset of the treatment. Green bars represent CT, and orange bars LTMH treatment. **(A)** PV of *def1*. LTMH consisted of repeated exposure to 30°C (day) and 24°C (night). Values indicate the mean ± SD (*n* = 5 plants, with multiple pools of 3 anther cones per plant). Two-way ANOVA main effects: temperature *P* < 0.01; genotype *P* < 0.001; interaction *P* < 0.01. **(B)** PV of *acx1*. LTMH consisted of repeated exposure to 31°C (day) and 25°C (night). Values indicate the mean ± SD (*n* = 5 plants, with multiple pools of 3 anther cones per plant). Two-way ANOVA main effects: temperature *P* < 0.001; genotype ns; interaction *P* < 0.05. *Significantly different between LTMH and CT in WT or between mutant and WT within temperature treatment (brackets), one-way ANOVA with LSD *post hoc*, *P* < 0.05; ^**^P < 0.01; ^***^P < 0.001.

Although the level of JA-ile was equally low in the wild type and low-SA line in LTMH, the lower expression of multiple JAZ repressor genes suggested that the transgenic line was more sensitive to JA. To evaluate the role of JA in the pollen LTMH phenotype, we assessed the JA biosynthesis mutants *defenseless1* (*def1*) and *acyl-CoA oxidase A1* (*acx1*), in which the JA level is reduced (Howe et al., 1996; Li et al., 2005). While both lines performed as well as the WT in CT, they had a significantly lower PV than WT already in very mild LTMH conditions (Figure 6). The hypersensitive response in LTMH suggests that a low JA signal may indeed limit pollen development in this condition.

## Discussion

In this study, we have shown that endogenous levels of SA, which is often associated with biotic and abiotic stress responses, negatively affect pollen LTMH tolerance. We found that reducing SA levels had distinct effects on anther and pollen physiology under LTMH, and found evidence for a role of JA signaling in the mechanism behind pollen thermotolerance of the low-SA line.

### Pollen Long-Term Mild Heat Tolerance Through Low Salicylic Acid Level

A high PV was observed in a *35S:nahG* low-SA tomato line compared to wild type when plants were subjected to LTMH. This result contrasts with the findings that SA has a positive effect on the basal and acquired thermotolerance of Arabidopsis seedlings exposed to heat shock (Larkindale et al., 2005; Clarke et al., 2009). Similarly, SA treatment reduced the effects of heat stress on the vegetative tissues of wheat (Khan et al., 2013), and rice spikelets (Zhang et al., 2017; Feng et al., 2018). A possible explanation is a difference in the degree of heat injury experienced by the plants. Formation of ROS has been reported at an absolute high temperature, while milder heat regimes, such as in LTMH conditions, do not lead to detectable ROS damage (Djanaguiraman et al., 2014, 2018; Zhao et al., 2018). Indeed, it has been proposed before that the positive effect of SA on thermotolerance is associated with increased ROS scavenging activity (Larkindale and Knight, 2002; Herrera-Vásquez et al., 2015; Feng et al., 2018). Furthermore, the positive effects shown by increasing the signal of SA are mostly derived from experiments on vegetative tissues. Increased levels of SA may cause a shift of resource allocation from growth and development to a stress acclimation response (Huot et al., 2014; Karasov et al., 2017; Bechtold and Field, 2018). While existing vegetative tissue may be able to endure a period of scarcity by entering into a state of metabolic quiescence, the highly dynamic process of pollen development may not be able to do so. Thus, there is the possibility that in LTMH conditions, the temperature itself may not be detrimental to pollen development, but rather the investment in stress tolerance pathways, which prepares the plant for more severe temperature conditions, is. Accordingly, SA treatment in non-stressed conditions may reduce PV (Praba and Thangaraj, 2005). From an evolutionary viewpoint, a strong early acclimation to be able to withstand further temperature increases at the organismal level may be a beneficial trade-off against a temporary reduction of PV. However, from an agricultural perspective, the same can be an undesirable trait as stress levels do not always become unsustainable.

### Low-Salicylic Acid Effects on Anther and Pollen Physiology in Long-Term Mild Heat

Based on transcriptomic and physiological analyses, several hypotheses for the mechanism behind higher pollen LTMH tolerance of the *35S:nahG* line might be posed. We found higher expression of photosynthesis-related gene expression in anthers of the low-SL line, counteracting the strong downregulation of these genes by LTMH in the wild type. Young anthers do contain chloroplasts for photosynthesis, which might be hypothesized to then function better in the low-SA line. However, it has been reported before that LTMH-type temperatures hardly affect photosynthesis rates (Camejo et al., 2005). Moreover, excluding light from developing flower buds did not compromise pollen development (data not shown). Thus, photosynthesis-related gene expression differences are not likely to be the reason for the pollen LTMH hypertolerance of the low-SA line.

There was a lower expression of mitosis-related genes in the low-SA anthers compared to wild type in LTMH specifically, indicating that certain cells in the low-SA anthers were less progressed toward mitosis, putatively the microspores. However, this process was not upregulated by LTMH in the wild type, suggesting it is not involved in the pollen LTMH phenotype. At the same time, there was decreased transcript accumulation of *TDF1*, *WBC27*, and *CYP703A2*, which are all expressed in the tapetum at early microspore stages (Xu et al., 2010; Xiong et al., 2016), and strongly repressed during further microspore development, suggesting advanced progression of certain tapetal developmental programs. Furthermore, we found that *MPK3* was expressed higher in the low-SA line. Knockout of *MPK3* in *Arabidopsis* results in smaller anthers, and in a double mutant with its closest homolog, *MPK6*, pollen development is severely impaired (Hord et al., 2008). The tomato orthologs of both, *MPK3* and *MPK6* (Solyc12g019460), were significantly downregulated by LTMH in the wild type; higher expression of *MPK3* in *35S:nahG* may thus prevent damage through this pathway. Taken together, various anther developmental pathways are differentially affected by *35S:nahG* in LTMH.

In addition, we found higher expression of *HSF6b* in LTMH and *HSF9* in CT and LTMH. It remains to be determined whether this constitutes an *HSFA2*-independent branch of the HSR that protects against LTMH. Enrichment analysis indicated that the responses to unfolded proteins and ER stress as a whole were reduced in *35S:nahG* in LTMH, which could be a result of constitutively better protection to protein unfolding. Carbohydrate-related gene annotations were overrepresented in the tolerant line. This was mainly caused by highly diverse expression changes of several *FBA*, *BAM*, *SWEET*, and *STP* genes, without a clear overall pattern. *35S:nahG* had a slightly increased sucrose and glucose level in bicellular and mature pollen stage anthers in LTMH, but overall carbohydrate levels were rather similar to wild type. Sucrose and hexose levels thus seem not to be predictive of pollen performance, either because they are not limiting at these observed levels, or because they do not sufficiently capture underlying metabolic fluxes.

### Enhanced Jasmonic Acid Signal May Mediate the Low-Salicylic Acid Effect on Pollen Long-Term Mild Heat Tolerance

Long-term mild heat had negative effects on the level of IAA, JA-ile, and iP in the wild type. When comparing the low-SA and wild-type plants, only the drop in IAA level seemed to be mitigated to some extent, raising the possibility that this contributed to improved LTMH tolerance. Indeed, indications that low IAA levels are causal for pollen failure have been reported for barley (Sakata et al., 2010). An absence of JA signal also causes severe anther and pollen developmental defects in Arabidopsis and reduced PV in tomatoes (Xie et al., 1998; Li et al., 2004). The severe pollen phenotype in two low-JA genotypes at mildly elevated temperature shows that there is a temperature-dependent requirement for this hormone in the process of pollen development. Interestingly, *35S:nahG* dampened the upregulation by LTMH of several JAZ genes, encoding repressors of the JA response (Chini et al., 2017), raising the possibility that low JA signaling is causal to pollen LTMH damage and that increased JA signaling strength in *35S:nahG* contributes to better pollen performance of this line. Indeed, Arabidopsis seedlings require JA signaling for heat tolerance, and heat-induced male sterility has been linked to reduced JA signal (Clarke et al., 2009; Khan et al., 2020; He et al., 2021). It is likely, however, that both, the LTMH pollen phenotype in the wild type and the increased tolerance in the low-SA line involve interaction between multiple hormones. In addition to JA, SA, auxin, and ABA have all been implicated in pollen development (Cardarelli and Cecchetti, 2014; Feng et al., 2018; Yao et al., 2018; Rezaul et al., 2019; Terceros et al., 2020). Furthermore, multiple hormones are associated with cell division (Shimotohno et al., 2021), carbohydrate metabolism (Eveland and Jackson, 2012), and the heat shock response (Snyman and Cronjé, 2008; Carranco et al., 2010; Huang et al., 2016; Tian et al., 2020; Li et al., 2021). Analysis of the role of single hormones as well as their interaction will be needed to be able to model and forecast the effects of hormone modifications on pollen heat tolerance.

Taken together, we have shown that low endogenous SA level promotes pollen development under LTMH conditions and found a number of plausible mechanisms for the tolerant phenotype. Future studies might investigate the involvement of specific genes, such as *MPK3* and *HSF*s, and the hormones IAA and JA, e.g., through analysis of lines with reduced JAZ gene activity. For potential future applications, it will be important to test whether anther-specific reduction of SA is sufficient to obtain the desired tolerance.

## Data Availability Statement

The datasets presented in this study can be found in online repositories. The names of the repository/repositories and accession number(s) can be found below: https://www.ncbi.nlm.nih.gov/geo/, GSE185583.

## Author Contributions

SJ and IR conceived and designed the study and wrote the manuscript. SJ, LS, YT, and WK performed the research. SJ, WL, and IR analyzed and interpreted the data. All authors contributed to the article and approved the submitted version.

## Conflict of Interest

The authors declare that the research was conducted in the absence of any commercial or financial relationships that could be construed as a potential conflict of interest.

## Publisher’s Note

All claims expressed in this article are solely those of the authors and do not necessarily represent those of their affiliated organizations, or those of the publisher, the editors and the reviewers. Any product that may be evaluated in this article, or claim that may be made by its manufacturer, is not guaranteed or endorsed by the publisher.

## Footnotes

1
https://biit.cs.ut.ee/clustvis_large/

2
http://www.pantherdb.org
