# Supplementary material for: Low Salicylic Acid Level Improves Pollen Development Under Long-Term Mild Heat Conditions in Tomato
Source: Front Plant Sci. 2022 Apr 11;13:828743. doi: 10.3389/fpls.2022.828743 (PMC9036445; doi:10.3389/fpls.2022.828743)
Supplement: Supplementary file 1 [file Image_1.PDF]

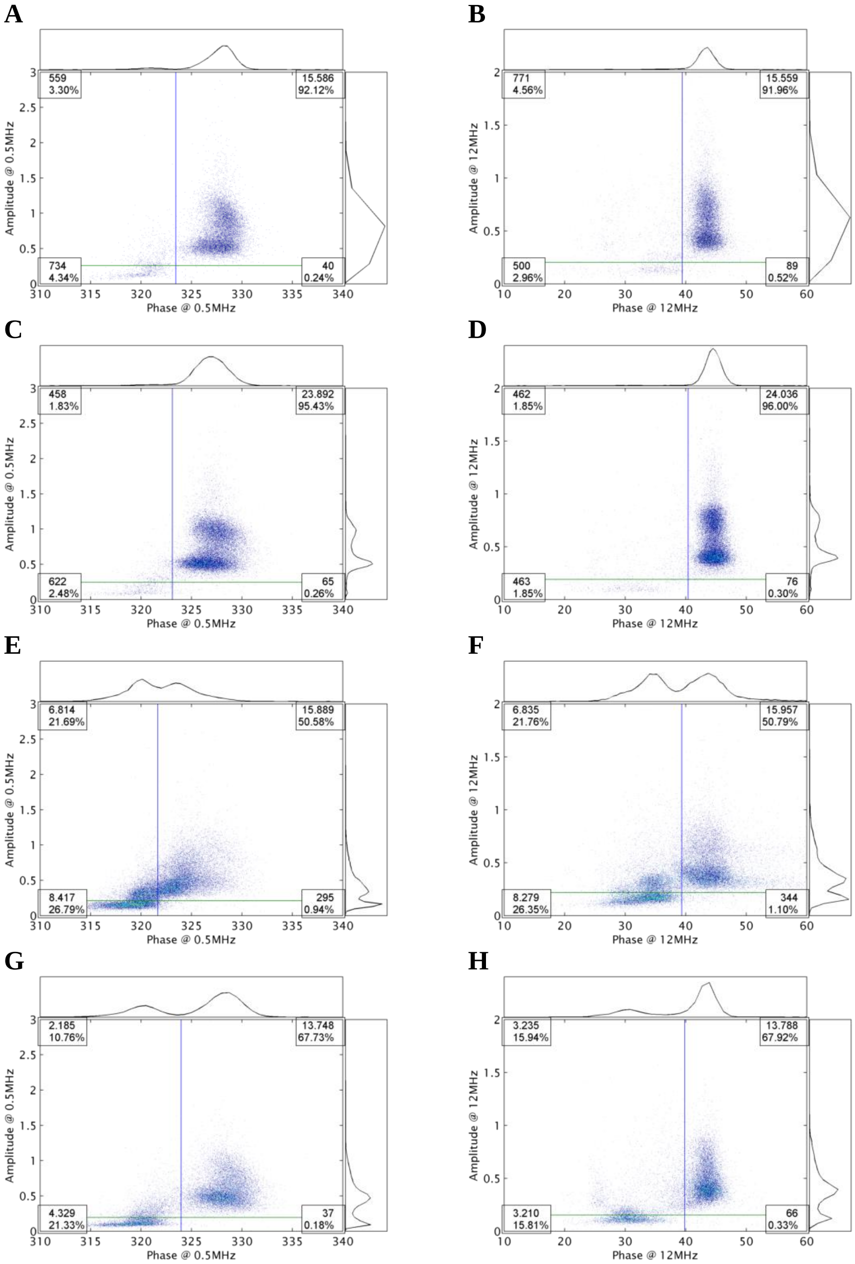

Supplementary Figure 1. Representative outputs of impedance flow cytometry pollen viability measurements. For each pollen the amplitude and phase change of the electrical resistance (impedance) of the running buffer is plotted, roughly corresponding to cell size and membrane potential, respectively (Heidmann et al., 2016). The datapoints in the upper right quadrant represent viable pollen, while the upper- and lower left quadrants contain data of non-viable pollen; the lower right quadrant may contain cell debris and other non-pollen contaminants. Pollen viability of wild-type in CT conditions **(A, B)**, *35S::nahG* in CT conditions **(C, D)**, wild-type in LTMH conditions **(E, F)** and *35S::nahG* in LTMH conditions **(G, H)**. **A, C, E, G)** Impedance to a frequency of 0.5 MHz, **B, D, F, H)** Impedance to a frequency of 12 MHz**.**
