# Supplementary material for: Low Salicylic Acid Level Improves Pollen Development Under Long-Term Mild Heat Conditions in Tomato
Source: Front Plant Sci. 2022 Apr 11;13:828743. doi: 10.3389/fpls.2022.828743 (PMC9036445; doi:10.3389/fpls.2022.828743)
Supplement: Supplementary file 2 [file Image_2.PDF]

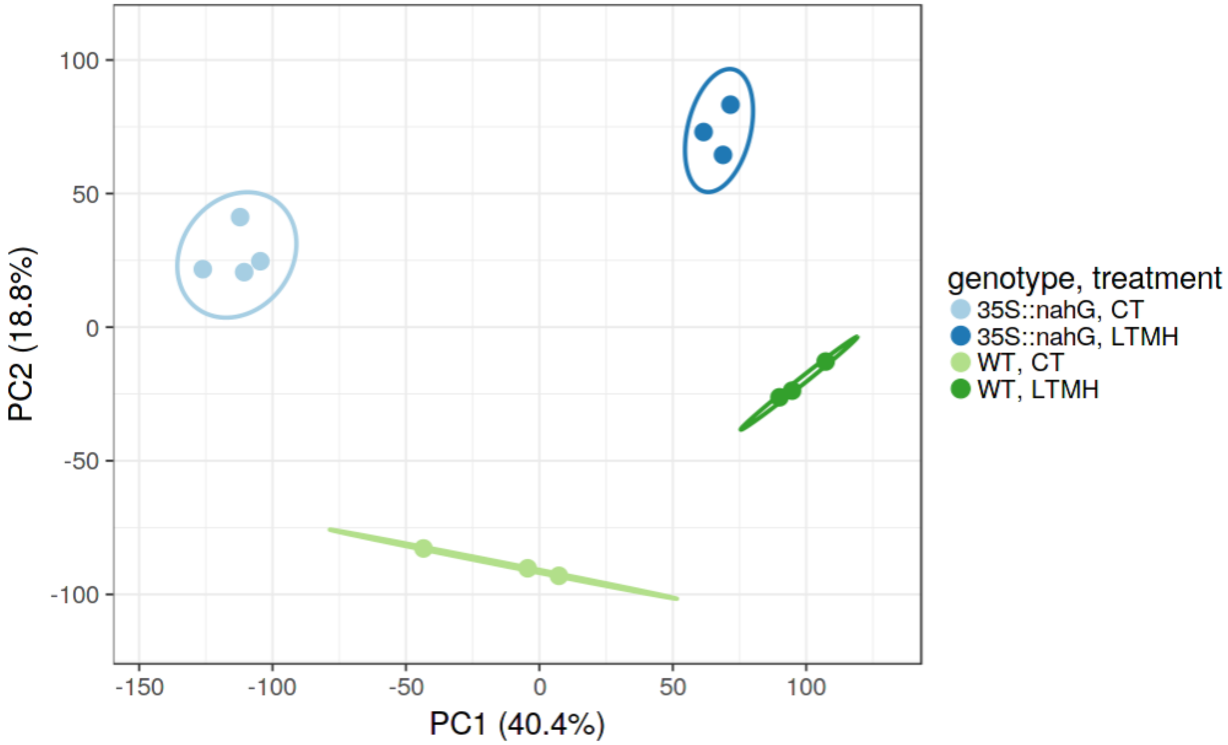

Supplementary Figure 2. Principal component analysis of transcriptome data from polarized microspore stage anthers of *35S::nahG* and wild type in CT and LTMH.
