# Supplementary material for: Low Salicylic Acid Level Improves Pollen Development Under Long-Term Mild Heat Conditions in Tomato
Source: Front Plant Sci. 2022 Apr 11;13:828743. doi: 10.3389/fpls.2022.828743 (PMC9036445; doi:10.3389/fpls.2022.828743)
Supplement: Supplementary file 3 [file Image_3.PDF]

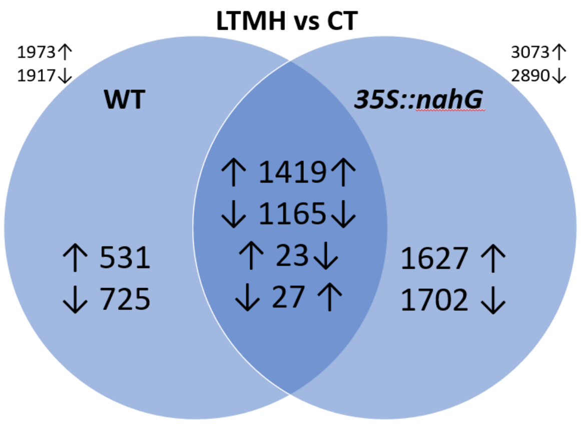

Supplementary Figure 3. Differentially expressed genes in polarized stage anthers of WT and *35S::nahG* between CT and LTMH. The Venn diagram represents DEGs of the LTMH-response, and the overlap between the two lines. LTMH consisted of repeated exposure to 34°C (day) and 28°C (night). Values indicate the number of DEGs, and up and down arrows indicate up- or downregulation, respectively, in LTMH. An arrow left to the value indicates the direction of response in WT; on the right, in *35S::nahG*. The values outside the diagram indicate the total number of DEGs for that genotype.
