# Supplementary material for: Low Salicylic Acid Level Improves Pollen Development Under Long-Term Mild Heat Conditions in Tomato
Source: Front Plant Sci. 2022 Apr 11;13:828743. doi: 10.3389/fpls.2022.828743 (PMC9036445; doi:10.3389/fpls.2022.828743)
Supplement: Supplementary file 4 [file Image_4.PDF]

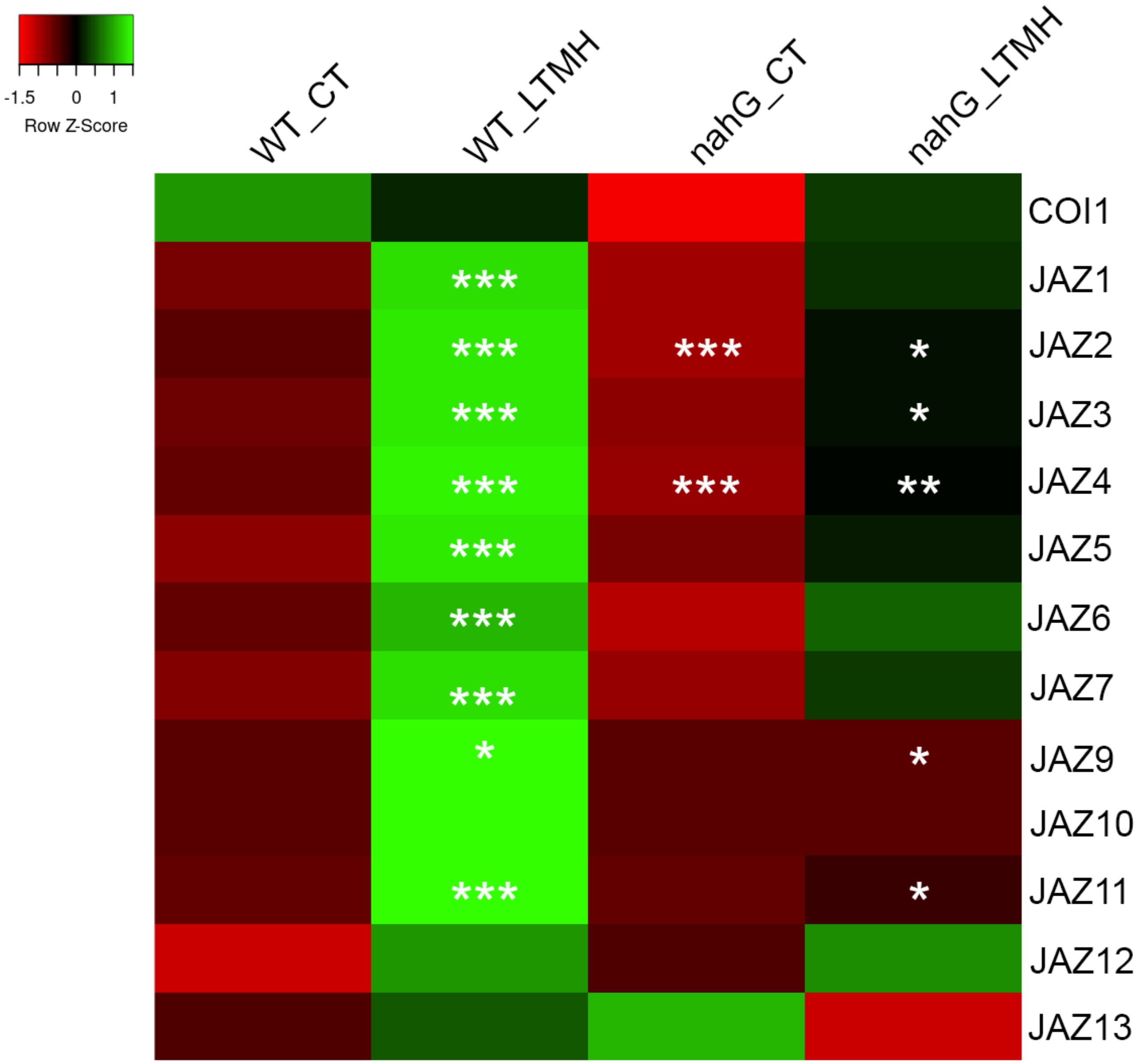

Supplementary Figure 4. Differential expression of *COI* and *JAZ* jasmonate signalling component genes between *35S::nahG* and WT. COI1, Solyc05g052620; JAZ1, Solyc07g042170; JAZ2, Solyc12g009220; JAZ3, Solyc03g122190; JAZ4, Solyc12g049400; JAZ5, Solyc03g118540; JAZ6, Solyc01g005440; JAZ7, Solyc11g011030; JAZ9, Solyc08g036640; JAZ10, Solyc08g036620; JAZ11, Solyc08g036660; JAZ12, Solyc01g009740; JAZ13, Solyc01g103600 (based on Chini et al., 2017). *, significantly different between LTMH and CT in WT or between mutant and WT within temperature treatment, |FC|>1.5, FDR q<0.05; **, FDR q <0.01; ***, FDR q <0.001.
