# Supplementary material for: Low Salicylic Acid Level Improves Pollen Development Under Long-Term Mild Heat Conditions in Tomato
Source: Front Plant Sci. 2022 Apr 11;13:828743. doi: 10.3389/fpls.2022.828743 (PMC9036445; doi:10.3389/fpls.2022.828743)
Supplement: Supplementary file 5 [file Image_5.PDF]

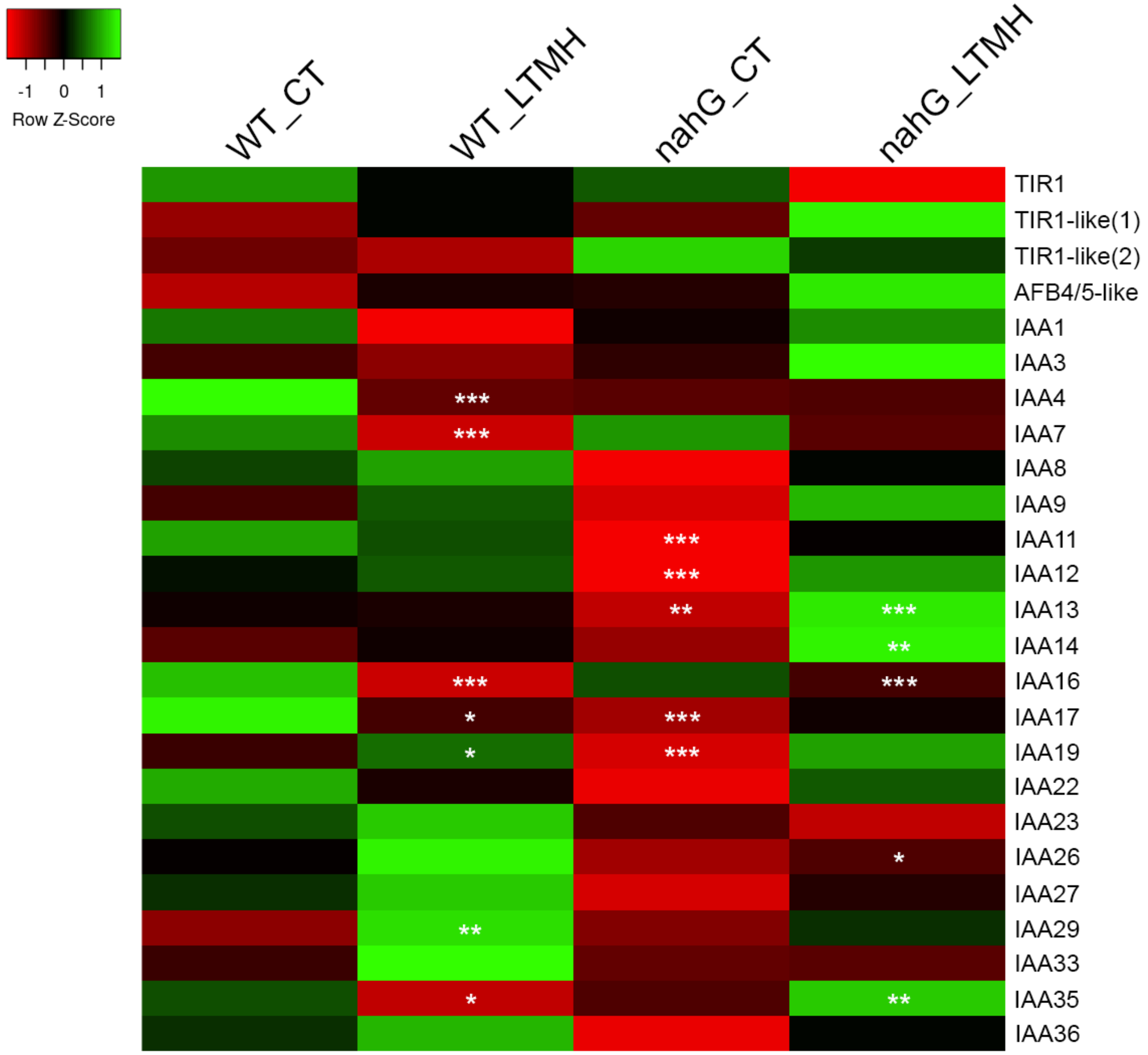

Supplementary Figure 5. Differential expression of *TIR1/AFB* and *AUX/IAA* auxin signalling components between *35S::nahG* and WT. TIR1, Solyc09g074520; TIR1-like(1), Solyc02g079190; TIR1-like(2), Solyc06g008780; AFB4/5, Solyc04g074980; IAA1, Solyc09g083280; IAA3, Solyc09g065850; IAA4, Solyc06g053840; IAA7, Solyc06g053830; IAA8, Solyc12g007230; IAA9, Solyc04g076850; IAA11, Solyc12g096980; IAA12, Solyc09g064530; IAA13, Solyc09g090910; IAA14, Solyc09g083290; IAA16, Solyc01g097290; IAA17, Solyc06g008590; IAA19, Solyc03g120380; IAA22, Solyc06g008580; IAA23, Solyc04g054280; IAA26, Solyc03g121060; IAA27, Solyc03g120500; IAA29, Solyc08g021820; IAA33, Solyc07g019450; IAA35, Solyc07g008020; IAA36, Solyc06g066020 (based on Audran-Delalande et al., 2012). *, significantly different between LTMH and CT in WT or between mutant and WT within temperature treatment, |FC|>1.5, FDR q<0.05; **, FDR q <0.01; ***, FDR q <0.001.
