# Supplementary material for: Low Salicylic Acid Level Improves Pollen Development Under Long-Term Mild Heat Conditions in Tomato
Source: Front Plant Sci. 2022 Apr 11;13:828743. doi: 10.3389/fpls.2022.828743 (PMC9036445; doi:10.3389/fpls.2022.828743)
Supplement: Supplementary file 7 [file Table_2.DOCX]

**Supplementary Table 2.** Flower length and sampling conditions for different stages of pollen development.

|  | **Polarized microspores** | **Binucleate pollen** | **Mature pollen** |
| --- | --- | --- | --- |
| DBA^1^ (days) | 7 | 4 | 1 |
| Flower length^2^ (mm) | 4.6-4.8 | 6.1-6.4 | 7.0-7.5 |
| Days HT (minimum) | 7 | 10 | 13 |
| Days HT (sampling) | 8 | 11 | 14 |
| # of anthers/200 mg | 65 | 40 | 25 |

^1^DBA: days before anthesis

^2^Flower length was measured including the receptacle, but excluding (protruding) sepals.
