# Supplementary material for: Low Salicylic Acid Level Improves Pollen Development Under Long-Term Mild Heat Conditions in Tomato
Source: Front Plant Sci. 2022 Apr 11;13:828743. doi: 10.3389/fpls.2022.828743 (PMC9036445; doi:10.3389/fpls.2022.828743)
Supplement: Supplementary file 8 [file Table_3.DOCX]

**Supplementary Table 3.** Multiple reactions monitoring (MRM) transitions table for all plant growth regulators and corresponding internal standards used in this study.

| **Number** | **Compound** | **Retention Time** | **Mass^1^** | **MRM transition** | **Cone V.** | **Coll. Energy** |
| --- | --- | --- | --- | --- | --- | --- |
| 1 | IAA | 3.34 | +176.25 | 103.2 | 30 | 25 |
|  |  |  |  | 130.2^‡^ | 30 | 15 |
| 2 | ^[13C6]^IAA | 3.32 | +182.1 | 109.2 | 30 | 25 |
|  |  |  |  | 136.2^‡^ | 30 | 15 |
| 3 | SA | 3.64 | -137.1 | 93.1^‡^ | 40 | 15 |
|  |  |  | +139.1 | 121.0 | 18 | 12 |
|  | ^[2H4]^SA | 3.60 | -141.1 | 97.1^‡^ | 40 | 15 |
|  |  |  | +143.1 | 124.9 | 20 | 14 |
| 4 | ABA | 4.15 | -263.25 | 153.15^‡^ | 30 | 10 |
|  |  |  |  | 219.15 | 30 | 15 |
| 5 | ^[2H6]^ABA | 4.12 | -269.25 | 159.15^‡^ | 30 | 10 |
|  |  |  |  | 225.15 | 30 | 15 |
| 9 | JA-Ile | 8.38 | +324.45 | 86.25^‡^ | 35 | 20 |
|  |  |  |  | 151.3 | 35 | 15 |
|  |  |  |  | 278.45 | 35 | 10 |
| 10 | ^[2H2]^JA-Ile | 8.35 | +326.4 | 151.3^‡^ | 35 | 15 |
|  |  |  |  | 280.45 | 35 | 10 |
| 11 | iP | 8.17 | 204.1 | 69.1 | 40 | 20 |
|  |  |  |  | 136.2^‡^ | 40 | 10 |
| 12 | ^[2H2]^iP | 8.14 | 210.1 | 75.08 | 40 | 20 |
|  |  |  |  | 137.1^‡^ | 40 | 10 |
| 13 | tZ | 2.84 | 220.3 | 136.25^‡^ | 40 | 15 |
|  |  |  |  | 148.25 | 40 | 15 |
|  |  |  |  | 202.3 | 40 | 10 |
| 14 | ^[2H3]^tZ | 2.80 | 225.3 | 137.25^‡^ | 40 | 15 |
|  |  |  |  | 148.25 | 40 | 15 |
|  |  |  |  | 207.25 | 40 | 10 |
| 15 | cZ | 3.34 | 220.3 | 136.25^‡^ | 40 | 15 |
|  |  |  |  | 148.25 | 40 | 15 |
|  |  |  |  | 202.3 | 40 | 10 |

^1^Mass in positive (+) or negative (-) ion mode.

^‡^Transition used for quantification
