# Supplementary material for: Low Salicylic Acid Level Improves Pollen Development Under Long-Term Mild Heat Conditions in Tomato
Source: Front Plant Sci. 2022 Apr 11;13:828743. doi: 10.3389/fpls.2022.828743 (PMC9036445; doi:10.3389/fpls.2022.828743)
Supplement: Supplementary file 10 [file Table_5.DOCX]

**Supplementary Table 5.** Panther gene-set enrichment analysis of gene expression differences between *35S::nahG* and WT in LTMHT conditions.

| **PANTHER GO-Slim Biological Process** | | **#** | **+/-** | **p-value** | **FDR** |
| --- | --- | --- | --- | --- | --- |
| GO:0015979 | photosynthesis | 36 | + | 2.20E-12 | 3.34E-09 |
| GO:0006270 | DNA replication initiation | 18 | - | 1.44E-05 | 1.46E-03 |
| GO:0009737 | response to abscisic acid | 33 | + | 4.00E-05 | 2.76E-03 |
| GO:0033260 | nuclear DNA replication | 13 | - | 1.49E-04 | 7.06E-03 |
| GO:0071897 | DNA biosynthetic process | 93 | - | 1.72E-04 | 7.92E-03 |
| GO:0006414 | translational elongation | 258 | - | 2.43E-04 | 8.81E-03 |
| GO:0035967 | cellular response to topologically incorrect protein | 51 | - | 2.87E-04 | 9.08E-03 |
| GO:0009611 | response to wounding | 15 | - | 4.27E-04 | 1.16E-02 |
| GO:0031347 | regulation of defence response | 19 | - | 6.18E-04 | 1.59E-02 |
| GO:0034976 | response to endoplasmic reticulum stress | 66 | - | 6.19E-04 | 1.57E-02 |
| GO:0046907 | intracellular transport | 476 | - | 7.90E-04 | 1.88E-02 |
| GO:0006302 | double-strand break repair | 89 | - | 8.82E-04 | 2.03E-02 |
| GO:0006091 | generation of precursor metabolites and energy | 148 | + | 9.56E-04 | 2.17E-02 |
| GO:0030490 | maturation of SSU-rRNA | 41 | - | 9.77E-04 | 2.15E-02 |
| GO:0000470 | maturation of LSU-rRNA | 36 | - | 1.19E-03 | 2.39E-02 |
| GO:1901566 | organonitrogen compound biosynthetic process | 652 | - | 1.56E-03 | 2.97E-02 |
| GO:0140014 | mitotic nuclear division | 198 | - | 1.58E-03 | 2.87E-02 |
| GO:0006271 | DNA strand elongation involved in DNA replication | 11 | - | 1.64E-03 | 2.89E-02 |
| GO:0015031 | protein transport | 373 | - | 1.96E-03 | 3.39E-02 |
| GO:0000027 | ribosomal large subunit assembly | 38 | - | 2.02E-03 | 3.38E-02 |
| GO:0071824 | protein-DNA complex subunit organization | 95 | - | 2.06E-03 | 3.41E-02 |
| GO:0031497 | chromatin assembly | 46 | - | 2.41E-03 | 3.89E-02 |
| GO:0000076 | DNA replication checkpoint | 13 | - | 2.44E-03 | 3.91E-02 |
| GO:0001932 | regulation of protein phosphorylation | 60 | - | 2.53E-03 | 4.00E-02 |
| GO:0009416 | response to light stimulus | 78 | + | 2.65E-03 | 4.16E-02 |
| GO:0009312 | oligosaccharide biosynthetic process | 28 | + | 2.79E-03 | 4.28E-02 |
| GO:0044248 | cellular catabolic process | 693 | - | 2.93E-03 | 4.37E-02 |
| GO:0006006 | glucose metabolic process | 28 | + | 2.98E-03 | 4.35E-02 |
