# Supplementary material for: Low Salicylic Acid Level Improves Pollen Development Under Long-Term Mild Heat Conditions in Tomato
Source: Front Plant Sci. 2022 Apr 11;13:828743. doi: 10.3389/fpls.2022.828743 (PMC9036445; doi:10.3389/fpls.2022.828743)
Supplement: Supplementary file 13 [file Table_8.DOCX]

**Supplementary Table 8.** Overrepresentation analysis of gene sets among genes differentially expressed between *35S::nahG* versus and type in LTMH conditions (pollen development, heat shock response (HSR), and carbohydrate metabolism and transport).

|  | **LTMH** | | | | | |
| --- | --- | --- | --- | --- | --- | --- |
|  | **Low-SA > WT** | | **Low-SA < WT** | | **Total** | |
| **pollen development (254)**^1^ |  | 6 |  | 9 |  | 15 |
| **HSR (219)** |  | 10 |  | 9 |  | 19 |
| **carbohydrates (210)** |  | 21^***2^ |  | 8 |  | 29^***^ |

^1^The number in brackets indicates the total amount of genes in the subset.

^2^Red shades indicate overrepresentation of gene sets.

*, significantly overrepresented as determined by Chi-square test with Yates’ correction, P < 0.05; **, P < 0.01; ***, P < 0.001.
