# Supplementary material for: Low Salicylic Acid Level Improves Pollen Development Under Long-Term Mild Heat Conditions in Tomato
Source: Front Plant Sci. 2022 Apr 11;13:828743. doi: 10.3389/fpls.2022.828743 (PMC9036445; doi:10.3389/fpls.2022.828743)
Supplement: Supplementary file 14 [file Table_9.DOCX]

**Supplementary Table 9.** Pollen, tapetum, and anther development-related genes that are significantly differentially expressed between *35S::nahG* and WT in LTMH.

|  | | **LTMH** | | **CT^1^** |
| --- | --- | --- | --- | --- |
| **GeneID** | **Gene** | **log_2_(FC)** | **FDR q** |  |
| Solyc06g005170 | MPK3 | 2.394 | 3.25E-57 |  |
| Solyc11g005985 | CALS5^3^ | 2.066 | 6.72E-03 |  |
| Solyc10g018780 | SlySBP8a | 0.843 | 1.57E-04 | ↑***^2^ |
| Solyc06g051750 | CPD | 0.833 | 3.30E-08 | ↑*** |
| Solyc08g042000 | SPS2F^3^ | 0.775 | 1.08E-06 | ↑*** |
| Solyc05g015390 | SRP1^3^ | 0.587 | 1.41E-14 | ↑*** |
| Solyc02g086920 | MTN3^3^ | -0.605 | 1.31E-03 | ↓*** |
| Solyc01g008820 | SSP^3^ | -0.621 | 1.88E-18 | ↑*** |
| Solyc11g066690 | RMI2^3^ | -0.722 | 4.57E-02 | ↓*** |
| Solyc05g007150 | UTR3^3^ | -0.747 | 1.65E-14 |  |
| Solyc05g052490 | APY7^3^ | -0.925 | 2.36E-03 |  |
| Solyc10g009390 | CYP703A2 | -3.349 | 2.44E-02 | ↑*** |
| Solyc01g010900 | CYP704B1 | -3.534 | 1.65E-02 | ↑*** |
| Solyc03g059200 | TDF1 | -4.441 | 1.13E-02 | ↓* |
| Solyc07g065780 | WBC27 | -5.225 | 6.08E-04 | ↑*** |

^1^ Difference between *35S::nahG* and WT in CT as reference.

^2^↑, upregulated in *35S::nahG*; ↓, downregulated; *, significantly differentially expressed between *35S::nahG* and WT in CT, P<0.05; ***, P<0.001.

^3^No *Solanum lycopersicum* annotation; closest *Arabidopsis* homolog is displayed.
