# Supplementary material for: Low Salicylic Acid Level Improves Pollen Development Under Long-Term Mild Heat Conditions in Tomato
Source: Front Plant Sci. 2022 Apr 11;13:828743. doi: 10.3389/fpls.2022.828743 (PMC9036445; doi:10.3389/fpls.2022.828743)
Supplement: Supplementary file 15 [file Table_10.DOCX]

**Supplementary Table 10.** Heat Shock Response-related genes that are significantly differentially expressed between *35S::nahG* and WT in LTMH.

|  | | | **LTMH** | | **CT^1^** |
| --- | --- | --- | --- | --- | --- |
| **GeneID** | **Gene** | **Family^2^** | **log_2_(FC)** | **FDR q** |  |
| Solyc04g081530 | DnaJ-C | HSP40 | 1.817 | 9.89E-03 | ↑***^3^ |
| Solyc06g053960 | HsfA6b | HSF | 1.744 | 6.39E-04 |  |
| Solyc06g053950 | HsfA6b | HSF | 1.488 | 5.83E-03 |  |
| Solyc07g040680 | HsfA9 | HSF | 1.315 | 2.50E-05 | ↑*** |
| Solyc04g063390 | DnaJ-C | HSP40 | 0.97 | 3.34E-02 | ↑*** |
| Solyc05g055160 | DnaJ-A | HSP40 | 0.85 | 9.80E-51 | ↑*** |
| Solyc03g115120 | DnaJ-C | HSP40 | 0.752 | 2.24E-02 |  |
| Solyc06g052050 | BIP2 | HSP70 | 0.681 | 2.44E-03 |  |
| Solyc01g096700 | DnaJ-C | HSP40 | 0.675 | 1.42E-32 | ↑*** |
| Solyc05g010240 | Cpn60-β (4) | HSP60 | 0.654 | 5.32E-06 |  |
| Solyc01g079610 | DnaJ-B | HSP40 | -0.634 | 1.91E-08 | ↑*** |
| Solyc09g007630 | DnaJ-C | HSP40 | -0.745 | 4.41E-09 | ↑*** |
| Solyc01g099660 | BIP3 | HSP70 | -0.758 | 4.64E-02 |  |
| Solyc04g016000 | HsfB3a | HSF | -0.925 | 1.29E-05 | ↑*** |
| Solyc07g043560 | Hsp70/110-ER | HSP70 | -0.963 | 1.83E-20 | ↑* |
| Solyc04g081570 | Hsp90-7 | HSP90 | -1.006 | 4.41E-73 | ↑*** |
| Solyc09g015000 | Hsp15.2-CI | sHsp | -5.323 | 4.91E-09 | ↓*** |
| Solyc09g018960 | DnaJ-C | HSP40 | -6.915 | 8.62E-44 | ↓*** |
| Solyc09g015020 | Hsp17.7A-CI | sHsp | -7.81 | 5.36E-09 | ↓*** |

^1^Difference between *35S::nahG* and WT in CT as reference.

^2^HSP, heat shock protein; HSF, heat shock factor; sHsp, small heat shock protein

^3^↑, upregulated in *35S::nahG*; ↓, downregulated; *, significantly differentially expressed between *35S::nahG* and WT in CT, P<0.05; ***, P<0.001.
