# Supplementary material for: Low Salicylic Acid Level Improves Pollen Development Under Long-Term Mild Heat Conditions in Tomato
Source: Front Plant Sci. 2022 Apr 11;13:828743. doi: 10.3389/fpls.2022.828743 (PMC9036445; doi:10.3389/fpls.2022.828743)
Supplement: Supplementary file 16 [file Table_11.DOCX]

**Supplementary Table 11.** Carbohydrate metabolism-related genes that are significantly differentially expressed between *35S::nahG* and WT in LTMH.

|  | | | **LTMH** | | **CT^1^** |
| --- | --- | --- | --- | --- | --- |
| **GeneID** | **Gene** | **Family**^2^ | **log_2_(FC)** | **FDR q** |  |
| Solyc10g085650 | Lin5b | CWIN | 1.289 | 2.33E-06 | ↓***^3^ |
| Solyc01g110360 | FBA1/2 | FBA | 1.288 | 5.00E-34 | ↑*** |
| Solyc12g099200 | INVINH1 / CIF1 | INVINH | 1.211 | 1.74E-06 | ↓* |
| Solyc11g042850 |  | FK | 1.094 | 6.28E-05 | ↑*** |
| Solyc04g071340 | CYFBP | FBP | 1.061 | 8.80E-24 | ↑*** |
| Solyc09g091030 | BAM1 | BAM | 1.024 | 9.79E-14 |  |
| Solyc10g086730 | CFBP1 | FBP | 0.847 | 1.81E-37 | ↑* |
| Solyc02g084440 | FBA1/2 | FBA | 0.778 | 5.66E-15 | ↑*** |
| Solyc08g042000 | SPSA2 | SPS | 0.775 | 1.08E-06 | ↑*** |
| Solyc02g062340 | FBA1/2 | FBA | 0.743 | 1.20E-30 | ↑* |
| Solyc03g083090 | SSI | SS | 0.719 | 4.34E-05 |  |
| Solyc07g042550 | SS3 | SUS | 0.67 | 1.03E-04 | ↑*** |
| Solyc03g006860 | FK1 | FK | 0.642 | 4.10E-03 |  |
| Solyc03g121680 |  | CWIN | -1.748 | 2.01E-04 | ↓*** |
| Solyc12g099190 | VIF | INVINH | -2.266 | 1.13E-14 | ↓*** |
| Solyc08g079080 | LIN9 | VIN | -3.162 | 2.47E-03 | ↓*** |

^1^Difference between *35S::nahG* and WT in CT as reference.

^2^CWIN, cell-wall invertase; FBA, fructose biphosphate aldolase; INVINH, invertase inhibitor; FK, fructokinase; FBP, fructose-1,6-bisphosphatase; BAM, beta-amylase; SPS, sucrose phosphate synthase; SS, starch synthase; SUS, sucrose synthase; VIN, vacuolar invertase.

^3^↑, upregulated in *35S::nahG*; ↓, downregulated; *, significantly differentially expressed between *35S::nahG* and WT in CT, P<0.05; ***, P<0.001.
