# Supplementary material for: Low Salicylic Acid Level Improves Pollen Development Under Long-Term Mild Heat Conditions in Tomato
Source: Front Plant Sci. 2022 Apr 11;13:828743. doi: 10.3389/fpls.2022.828743 (PMC9036445; doi:10.3389/fpls.2022.828743)
Supplement: Supplementary file 17 [file Table_12.DOCX]

**Supplementary Table 12.** Carbohydrate transport-related genes that are significantly differentially expressed between *35S::nahG* and WT in LTMH.

|  | | | **LTMH** | | **CT^1^** |
| --- | --- | --- | --- | --- | --- |
| **GeneID** | **Gene** | **Family**^2^ | **log_2_(FC)** | **FDR q** |  |
| Solyc08g080300 | SlSTP6 | STP | 4.096 | 1.88E-03 |  |
| Solyc03g114200 | SlSWEET5a | SWEET | 3.82 | 1.58E-06 |  |
| Solyc11g017010 | SlSUT1 | SUT | 1.279 | 3.23E-02 |  |
| Solyc03g093410 | SlSTP16 | STP | 1.189 | 6.52E-04 | ↑*^3^ |
| Solyc01g080680 | SlSFP3 | SFP | 1.007 | 1.20E-02 |  |
| Solyc11g012450 | SlINT4 | INT | 0.952 | 4.98E-07 |  |
| Solyc01g008240 | SlSTP9 | STP | 0.61 | 3.23E-11 |  |
| Solyc02g005180 | SlSFP2 | SFP | 0.602 | 2.95E-03 | ↓*** |
| Solyc02g086920 | SlSWEET6a | SWEET | -0.605 | 1.31E-03 | ↓*** |
| Solyc08g082770 | SlSWEET7a | SWEET | -0.644 | 6.87E-22 | ↓*** |
| Solyc05g024260 | SlSWEET12c | SWEET | -1.041 | 1.42E-03 |  |
| Solyc04g064630 | SlSWEET1c | SWEET | -1.313 | 4.51E-04 |  |
| Solyc09g074530 | SlNEC1 | SWEET | -1.924 | 3.45E-64 | ↓*** |

^1^Difference between *35S::nahG* and WT in CT as reference.

^2^ STP, sugar transporter protein; SUT, sucrose transporter; SFP, sugar facilitator protein; INT, inositol transporter.

^3^↑, upregulated in *35S::nahG*; ↓, downregulated; *, significantly differentially expressed between *35S::nahG* and WT in CT, P<0.05; ***, P<0.001.
