# Supplementary material for: Low Salicylic Acid Level Improves Pollen Development Under Long-Term Mild Heat Conditions in Tomato
Source: Front Plant Sci. 2022 Apr 11;13:828743. doi: 10.3389/fpls.2022.828743 (PMC9036445; doi:10.3389/fpls.2022.828743)
Supplement: Supplementary file 18 [file Table_13.DOCX]

**Supplementary Table 13.** Overrepresentation of hormone-related gene sets in *35S::nahG* in LTMH conditions.

|  | **LTMH** | | | | | | |
| --- | --- | --- | --- | --- | --- | --- | --- |
|  | **Low-SA > WT** | | **Low-SA < WT** | | **Total** | | |
| **ABA metabolism (17)**^1^ |  | 1 |  | 0 |  | 1 | (522) 41^3^ |
| **ABA signalling (139)** |  | 7 |  | 2 |  | 9 |  |
| **ABA response (370)** |  | 20^**2^ |  | 12 |  | 32^*^ |  |
| **auxin metabolism (30)** |  | 1 |  | 0 |  | 1 | (521) 31 |
| **auxin transport (99)** |  | 4 |  | 3 |  | 7 |  |
| **auxin signalling (131)** |  | 9^*^ |  | 3 |  | 12 |  |
| **auxin response (311)** |  | 8 |  | 8 |  | 16 |  |
| **brassinosteroid metabolism (56)** |  | 2 |  | 1 |  | 3 | (154)  8 |
| **brassinosteroid signalling (53)** |  | 1 |  | 1 |  | 2 |  |
| **brassinosteroid response (47)** |  | 3 |  | 0 |  | 3 |  |
| **cytokinin metabolism (31)** |  | 1 |  | 3 |  | 4 | (161) 16 |
| **cytokinin signalling (37)** |  | 2 |  | 2 |  | 4 |  |
| **cytokinin response (93)** |  | 3 |  | 5 |  | 8 |  |
| **ethylene metabolism (36)** |  | 5^***^ |  | 1 |  | 6^*^ | (189) 10 |
| **ethylene signalling (58)** |  | 0 |  | 1 |  | 1 |  |
| **ethylene response (96)** |  | 3 |  | 1 |  | 4 |  |
| **gibberellin metabolism (44)** |  | 1 |  | 2 |  | 3 | (122)  7 |
| **gibberellin signalling (3)** |  | 0 |  | 0 |  | 0 |  |
| **gibberellin response (75)** |  | 1 |  | 3 |  | 4 |  |
| **jasmonate metabolism (70)** |  | 4 |  | 3 |  | 7 | (303) 28 |
| **jasmonate signalling (67)** |  | 2 |  | 5 |  | 7 |  |
| **jasmonate response (177)** |  | 7 |  | 7 |  | 14 |  |
| **salicylic acid metabolism (29)** |  | 1 |  | 1 |  | 2 | (227)  7 |
| **salicylic acid signalling (17)** |  | 0 |  | 1 |  | 1 |  |
| **salicylic acid response (182)** |  | 3 |  | 3 |  | 6 |  |

^1^The number in brackets indicates the total number of genes in the subset.

^2^Red shades indicate overrepresentation of gene sets.

^3^Summary per hormone, with the total number of genes in the set between brackets and total number of differentially expressed genes.

*, significantly overrepresented as determined by Chi-square test with Yates’ correction, P < 0.05; **, P < 0.01; ***, P < 0.001.
